# Stochastic Analysis of the SOS Response in *Escherichia coli*

**DOI:** 10.1371/journal.pone.0005363

**Published:** 2009-05-08

**Authors:** Yishai Shimoni, Shoshy Altuvia, Hanah Margalit, Ofer Biham

**Affiliations:** 1 Department of Molecular Genetics and Biotechnology, Faculty of Medicine, The Hebrew University, Jerusalem, Israel; 2 Racah Institute of Physics, The Hebrew University, Jerusalem, Israel; Center for Genomic Regulation, Spain

## Abstract

**Background:**

DNA damage in *Escherichia coli* evokes a response mechanism called the SOS response. The genetic circuit of this mechanism includes the genes *recA* and *lexA*, which regulate each other via a mixed feedback loop involving transcriptional regulation and protein-protein interaction. Under normal conditions, *recA* is transcriptionally repressed by LexA, which also functions as an auto-repressor. In presence of DNA damage, RecA proteins recognize stalled replication forks and participate in the DNA repair process. Under these conditions, RecA marks LexA for fast degradation. Generally, such mixed feedback loops are known to exhibit either bi-stability or a single steady state. However, when the dynamics of the SOS system following DNA damage was recently studied in single cells, ordered peaks were observed in the promoter activity of both genes (Friedman et al., 2005, PLoS Biol. 3(7):e238). This surprising phenomenon was masked in previous studies of cell populations. Previous attempts to explain these results harnessed additional genes to the system and deployed complex deterministic mathematical models that were only partially successful in explaining the results.

**Methodology/Principal Findings:**

Here we apply stochastic methods, which are better suited for dynamic simulations of single cells. We show that a simple model, involving only the basic components of the circuit, is sufficient to explain the peaks in the promoter activities of *recA* and *lexA*. Notably, deterministic simulations of the same model do not produce peaks in the promoter activities.

**Conclusion/Significance:**

We conclude that the double negative mixed feedback loop with auto-repression accounts for the experimentally observed peaks in the promoter activities. In addition to explaining the experimental results, this result shows that including additional regulations in a mixed feedback loop may dramatically change the dynamic functionality of this regulatory module. Furthermore, our results suggests that stochastic fluctuations strongly affect the qualitative behavior of important regulatory modules even under biologically relevant conditions, thus emphasizing the importance of stochastic analysis of regulatory circuits.

## Introduction

**Figure 1:**
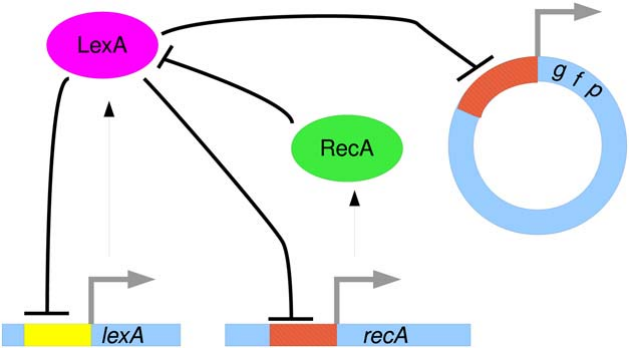
Schematic diagram of the *lexA*-*recA* circuit and the reporter gene used in Ref. [11]. LexA is a transcriptional regulator that represses its own transcription and that of *recA*. Following DNA damage, RecA negatively regulates the activity of LexA by protein-protein interaction. The flat-headed arrows represent negative regulation. The fluorescent reporter plasmid, used for the measurement of promoter activity, is also shown. This plasmid includes a promoter region identical to that of *recA*.

*Escherichia coli* cells respond to DNA damage by invoking a repair mechanism called the SOS response [1]–[5]. This mechanism encompasses a few dozen genes, most of which are regulated by the transcriptional repressor LexA, which is also an auto-repressor. Among these is the *recA* gene, which plays a major role in DNA repair, and also reduces the expression levels of *lexA* by an interaction between their protein products. Thus, *lexA* and *recA* define a double-negative mixed feedback loop that is at the heart of the SOS response. Under normal conditions the repressor LexA represses the transcription of several genes involved in DNA damage repair, keeping the transcription of these genes at a basal level. DNA damage from ultra-violet (UV) irradiation is manifested mainly by lesions in the DNA. This results in stalling of the DNA polymerase (Pol III) replication fork, and in the production of stalled single stranded DNA (ssDNA). The protein RecA binds to the stalled ssDNA [1]–[5]. RecA, along with other proteins, allows the replication fork to continue replication using homologous recombination [5]–[9]. Furthermore, when RecA is bound to the ssDNA, it becomes an active catalyst for the cleavage of the transcriptional repressor LexA [10], lowering the level of LexA and relieving the repression of the genes required for the damage repair, including its own transcription and that of *lexA* (see Fig. 1 for a schematic diagram).

Recently, single cell measurements of the SOS system have revealed intriguing dynamical properties [11]. This was done using fluorescent reporter genes that were inserted on plasmids into *E. coli* cells in order to measure the promoter activities of several genes involved in the SOS system. It was found that after UV irradiation, the promoter activities of both *recA* and *lexA* increase after a short delay, and reach peak values after about 30 minutes. If the irradiation is sufficiently strong, a second peak appears after 60–80 minutes and a third peak appears after 90–130 minutes. This result was somewhat puzzling, as usually double-negative mixed feedback loops, such as the one defined by *lexA* and *recA*, exhibit either bi-stability or a single steady state. Friedman et al. [11] explained this interesting dynamics by the involvement of the umuDC product, for which they provided experimental support. Subsequently, the dynamics of several of the molecules involved in the SOS network was studied using rate equations and the functional role of each regulation process was identified [12]. In particular, the second peak in the activity of *recA* and *lexA* was attributed to a positive feedback loop in which Pol V activates RecA filaments. This rather complex model, did not, however, succeed to explain the properties observed experimentally of the second and third peaks. Recently it was shown that including approximately twenty additional processes, and using stochastic simulations, it is possible to reproduce the experimental results after fitting many unknown parameters [13].

In this paper we reproduce the peaks in promoter activity, using stochastic simulations that follow the experimental procedure carried out in Ref. [11]. To this end we present a rather simple model that includes only the basic components of the system, *recA* and *lexA*, and their mutual regulations. Thus, this small sub-network that includes both a double-negative mixed feedback loop and auto-repression is sufficient for explaining the peaks in promoter activity. These results, obtained using stochastic simulations, are qualitatively different from those of deterministic methods. They demonstrate the importance of stochastic methods for understanding the dynamics of molecular mechanisms at a single cell level.

## Results

The experimental measurements of the expression dynamics of various genes in the SOS system enable re-examination of the known regulatory network. If under the experimental conditions, the system can be considered as an independent module, one expects the mathematical model to reproduce the observed dynamics. Based on the experimental results, a failure to reproduce the dynamics may be due to one of three possibilities: (a) the sub-network that was modeled is too small; (b) additional unknown regulations exist in the system; or (c) the methodology used for the dynamic simulations was inadequate. Our aim is therefore to identify the core sub-network of the SOS regulation network, and develop a mathematical model that reproduces the peaks observed experimentally in Ref. [11]. To this end we consider a small sub-network, which consists of the two genes *recA* and *lexA* and their mutual regulations. These two genes form a feedback loop which is essential for identifying and stabilizing the internal state of the system [14]. In this sub-network, the *lexA* gene codes for LexA proteins which act as transcriptional auto-repressors and as transcriptional repressors of *recA*. Under conditions of DNA damage, RecA proteins recognize this damage by polymerizing on stalled single stranded DNA (ssDNA). In this form, RecA proteins promote auto-cleavage of LexA proteins, thus acting as post translational repressors. A schematic diagram of this circuit is shown in Fig. 1. For simplicity, we assume that the degradation rate of RecA proteins does not depend on its binding to ssDNA. Furthermore, we assume that the DNA damage is sufficiently large so that virtually all RecA proteins are in their active form, inducing LexA auto-cleavage.

When there is no DNA damage, the LexA repressors ensure that both *recA* and *lexA* are expressed at low basal levels. When DNA damage occurs, the few RecA proteins that are present in the cell promote the cleavage of LexA proteins and lower their copy number. In case that the DNA damage persists, the negative regulation acting on both *recA* and *lexA* is eventually removed, and the promoter activity of both genes increases. This accounts for the delayed increase in promoter activity, and suggests that this increase depends on a threshold amount of DNA damage. When the number of *lexA* mRNA molecules reaches some threshold value, the production of LexA proteins becomes too fast for RecA to mark every LexA molecule for cleavage. As a result, the number of free LexA repressors increases and the promoter sites of *recA* and *lexA* quickly become occupied. The promoter activity of both genes is suppressed and their expression level decreases to the basal level. If the DNA damage is still present, RecA can further down-regulate LexA until its copy number drops to nearly zero. The system is now ready for the next burst of promoter activity. Such bursts will continue to appear as long as the DNA damage exists (provided that the cell remains alive). It should be noted that the only way to reduce the number of LexA to zero and initiate a burst of promoter activity is by means of stochastic fluctuations. When deterministic analysis in used, the expression levels of both genes reach some steady state value and do not exhibit any further change.

**Figure 2:**
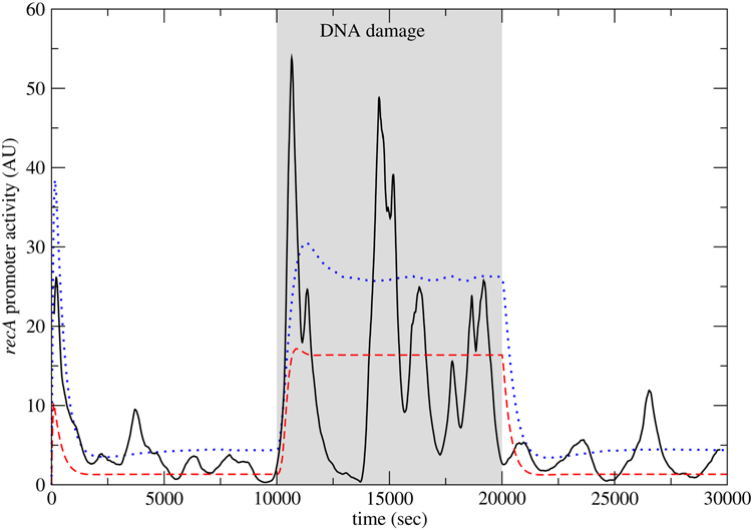
The promoter activity of *recA* vs. time. The results were obtained from the rate equations (dashed lines), from Monte Carlo simulations (solid lines), and from averaging over 10000 Monte Carlo simulations (dotted line). DNA damage is initiated at time 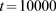 (sec) and removed at 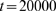 (sec). The rate equations do not predict any bursts in promoter activity following DNA damage. The Monte Carlo results show bursts in promoter activity separated by time intervals of low activity, but this behavior is masked when averaging over cell populations.

In Fig. 2 we show the promoter activity of *recA* vs. time, as obtained from rate equations (dashed line) and by a Monte Carlo simulation (solid line). DNA damage is introduced at time 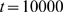 (sec) and repaired at 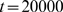 (sec). The rate equation results show no bursts in promoter activity as a result of the DNA damage. In contrast, the Monte Carlo results exhibit three large bursts during DNA damage, separated by intervals of low activity. Before and after DNA damage the promoter activity of *recA* is low, yet it exhibits some fluctuations. These fluctuations are due to the low copy number of LexA, which is an auto-repressor. The short initial rise in promoter activity in the rate equation model (in the first 1000 seconds) is consistent with the activation of an auto-repressor [15], which is known to “overshoot” its steady state. Additionally, Fig. 2 shows the average promoter activity as obtained from 10000 Monte Carlo simulations (dotted line). The results show that averaging over a population masks the bursting behavior seen in single cells, which is consistent with experimental results.

**Figure 3:**
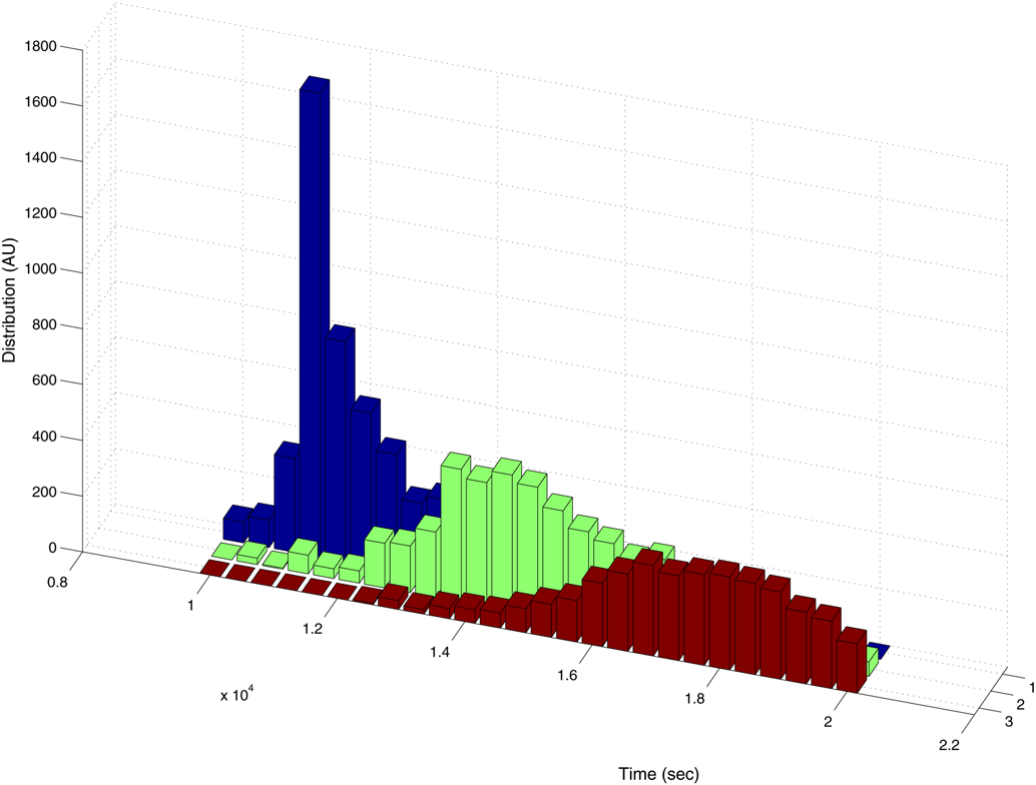
Probability distributions of the timings of the first, second and third peak in the promoter activity of *recA* following DNA damage. Three distinct distributions are observed, indicating that there is some level of regularity in the process. The results enable to identify the delay time between consecutive peaks, of about 3,000 (s). The results were obtained from approximately 9000 Monte Carlo simulations of the system.

To examine the regularity of the peaks, we analyzed the results of approximately 9000 Monte Carlo simulations. For each simulation we recorded the time of the first, second and third peak and formed a probability distribution, shown in Fig. 3. It can be seen that each of the three peaks appears within a well defined time interval following DNA damage. This demonstrates that the peaks are ordered. Using the specific set of parameters chosen in this simulation, the typical time between consecutive peaks is about 3000 (sec). The spread in peak timing in the second and third peaks stems from the stochastic nature of the system, and from the technical difficulty of distinguishing a local peak from a global one.

**Figure 4:**
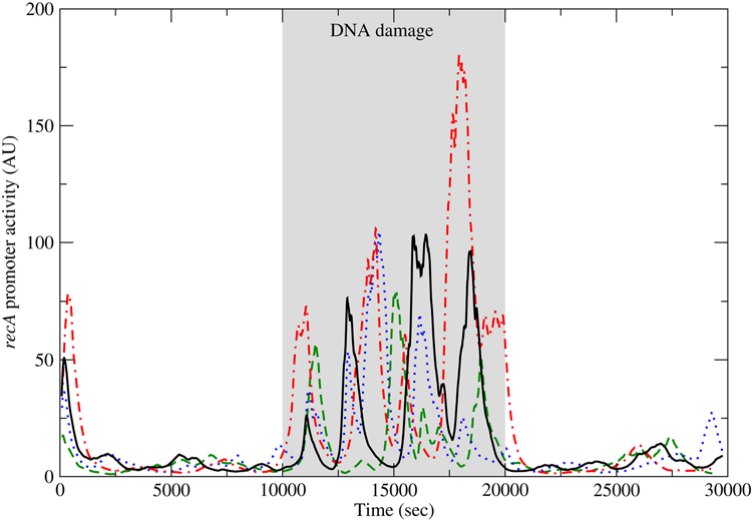
The promoter activity of *recA* vs. time, obtained by four Monte Carlo simulations. DNA damage is initiated at time 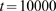 (sec) and removed at 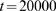 (sec). The four simulations show a simultaneous first peak in promoter activity. This synchronization is lost in subsequent peaks due to the stochastic nature of the system.

Further analysis of the timing of the peaks across cells is obtained by overlaying the simulation results of four Monte Carlo simulations, shown in Fig. 4. In all four simulations, after DNA damage, the first peak occurs after approximately 1000 seconds. All the cells proceed to display bursts in the promoter activity, but there is little synchronization between the cells due to the stochastic nature of the system.

The rate constants that are used in the simulations fall within the biologically relevant range. Specifically, to enable the peaks, the degradation rates of both RecA proteins and *recA* mRNA must be high (close to the upper limit of what is considered the biologically relevant range), so that the transcriptional regulation by LexA can affect the RecA expression quickly. Additionally, the total translation rate of LexA proteins at full promoter activity must be sufficiently high to eventually overcome the repression by RecA proteins. The values of the rate constants that were used in the simulation are presented in Table 1. An xml file of the model can be sent upon request. It should be noted that under the constraints laid out above, the ordered peaks in promoter activity are quite robust. Naturally, changes in specific parameters result in changes in the amplitude or in the characteristic time between the peaks. For example, a higher transcription rate or a lower degradation rate for the RecA proteins will result in a slightly larger amplitude and a longer period. We reached this conclusion after a scan of the parameter range which is biologically relevant (according to literature), and also complies with these constraints. It should also be noted that since there are many parameters in the model, a better fit of the simulations to the experimental results would not uniquely constrain all the parameters. When more of these parameters will be measured experimentally, it will become possible to constrain the rest of them by simulations. It will then also be possible to test the validity of the model in greater detail.

**Table 1:** Processes in the SOS system and their rates (sec^−1^).

Description	Rate Const.	Rate	Value
*recA* mRNA transcription ( 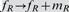 )	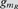	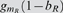	0.05
*lexA* mRNA transcription ( 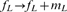 )	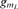	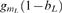	0.03
RecA protein translation ( 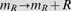 )	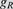	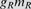	0.04
LexA protein translation ( 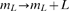 )	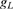	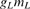	0.1
*recA* mRNA degradation ( 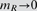 )	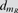	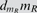	0.02
*lexA* mRNA degradation ( 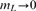 )	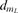	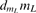	0.003
RecA protein degradation ( 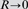 )	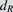	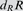	0.02
LexA protein degradation ( 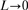 )	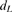	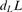	0.002
LexA binding to *recA* ( 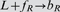 )	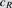	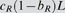	0.02
LexA binding to *lexA* ( 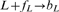 )	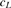	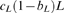	0.01
LexA dissociation from *recA* ( 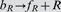 )	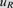	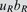	0.04
LexA dissociation from *lexA* ( 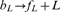 )	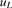	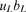	0.01
RecA marks LexA for cleavage ( 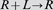 )	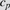	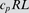	0.05
Reporter gene mRNA transcription ( 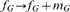 )	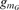	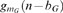	0.05
GFP translation ( 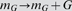 )	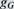	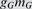	0.04
Reporter gene mRNA degradation ( 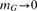 )	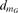	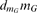	0.003
LexA binding to reporter gene ( 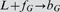 )	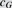	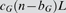	0.02
LexA dissociation from reporter gene ( 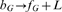 )	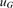	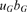	0.04

## Discussion

The sub-network of the SOS response system considered here consists of two feedback loops. The first is a negative auto-regulation feedback loop of *lexA*, and the second is a double-negative mixed feedback loop consisting of both *lexA* and *recA* (resulting in positive feedback of each gene to itself). This small sub-network can be considered as a separate module since it involves the only known feedback loop in the SOS network. It is possible, however, that adding more genes into the analysis will allow the results to fit the experimental results more closely. Similarly, it is likely that accounting for the time-delay in transcription and translation will also contribute to the robustness of the oscillatory behavior.

Both the feedback loops in the sub network are well studied network modules. The auto-repressor is a statistically significant network motif [16], [17], namely it appears in actual transcriptional regulatory networks much more often than expected in a random network [18]. It was proposed that the role of the auto-repressor is to speed up response times [19] and to reduce fluctuations and noise [20], [21]. The auto-repressor also tends to enhance the response to variations in the external conditions, before converging to a new steady state [15]. Using stochastic analysis it was shown that auto-regulatory feedback loops can produce small bursts of activity. These small bursts of activity can be seen both before and after the DNA damage in Fig. 2 and Fig. 4. They are, however, in general of much smaller magnitude than the bursts seen during DNA damage. Other negative feedback loops are known to produce oscillations, but this normally requires some delay between the regulation and its effect. In the auto-repressor loop the delay is not sufficient for producing oscillations.

The double-negative mixed feedback loop was also found to be a statistically significant network motif [22], and was shown to exhibit bi-stability for a broad range of parameters [23], [24]. When the feedback loop is bi-stable, in each of the stable states one of the genes is highly expressed and the other is suppressed. It should be noted that there are parameter domains in which only one of the two states is stable. In technical terms, feedback loops tend to lead to the creation of attractors (in phase-space), and in the case of a double negative feedback loop there may be either one or two attractors [18], [25].

Using the set of parameters that was used in this paper, the mixed feedback loop alone would have a single steady state in which the transcription factor LexA is highly expressed. However, the auto-repression of *lexA* makes this state unstable. Under these conditions, deterministic analysis using rate equations shows a single steady state, in which both genes are expressed at a low basal level. Stochastic analysis shows bursts of promoter activities of both genes, separated by time intervals of very low activity. The bursts can be viewed as resulting from the combination of the two feedback loops. The negative auto-regulation feedback loop tends to produce oscillations but lacks sufficient delay, and the coherent mixed feedback loop provides the necessary delay. Another way in which the two feedback loops may interacts was recently suggested, by showing that combining positive and negative feedbacks results in oscillations with a robust amplitude and varying frequency [26].

It should be noted that the simulations presented here do not replicate every aspect of the experimental results in Ref. [11]. This is likely due to the fact that the simulations consider only two genes in a large network of gene regulation. It is possible that taking into account the explanations put forward by Ref. [11], and Ref. [12] will allow a more detailed agreement between the simulation and the experiments. Specifically, this would mean the addition of two feedback mechanisms to the mathematical model, one mediated by UmuDC, and the other mediated by Pol V. However, since the rate constants involved in these processes are mostly unknown, such simulations will not contribute much to our knowledge of the system.

Our analysis suggests a set of experiments that can be performed in order to test the origin of the bursts in promoter activity. In such experiments, one of the regulatory feedback loops is disconnected. According to our model, this would produce a response to UV irradiation which does not exhibit bursts in promoter activity. For example, if the negative regulation of LexA on *recA* is removed, then the *recA* promoter should exhibit sustained elevated promoter activity, which will decrease only upon the resolution of all DNA damage.

The results presented above emphasize the importance of stochastic analysis of biological systems in which some of the participating entities appear in small copy numbers. Stochastic analysis can be used to study various models and reliably test to what extent they reproduce the experimental results. We conclude that the deterministic analysis alone is not sufficient in order to confirm or refute the validity or completeness of a given regulation network. Furthermore, these results suggest that when predicting the dynamics of regulatory systems, stochastic rather than deterministic analysis must be used.

## Methods

We have performed deterministic analysis of the SOS system using rate equations and stochastic analysis using Monte Carlo simulations based on the Gillespie algorithm [27]. These simulations enable to follow the copy number of each molecule in a single cell as a function of time. We follow the experimental procedure as presented in Ref. [11]. In this experiment, a GFP reporter gene was inserted into *E. coli* on a plasmid that carried the same promoter site as *recA*. These bacteria were then irradiated by UV, causing DNA damage. The amount of GFP was measured in single cells vs. time and statistical analysis of the results was performed.

To follow the experimental procedure we define the variables in the mathematical model describing the system in a single cell. The number of reporter genes in a single cell is denoted by 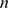. We denote the number of *recA* mRNAs, *lexA* mRNAs, and reporter gene mRNAs per cell by 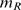, 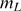, and 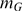, respectively. We denote the number of RecA, free LexA (unbound to any promoter site), and GFP proteins per cell by 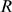, 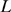, and 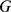, respectively. The number of LexA proteins that are bound to the promoter site of *recA*, *lexA*, and the reporter genes are denoted by 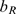, 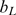, and 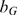, respectively. For convenience, we also use the number of unbound (free) promoters for each gene, denoting them by 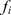, 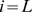, 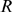 or 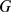 (e.g. 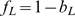). The rate constant for the transcription of *recA* mRNAs, *lexA* mRNAs, and the reporter gene mRNAs are denoted by 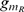, 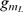, and 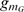, respectively. The translation rate constants of the RecA, LexA, and GFP proteins are denoted by 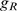, 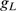, and 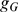, respectively. The degradation rate constants are denoted by 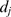, where 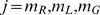 for mRNA molecules and 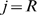 or 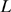 for proteins. The rate constant for the binding of LexA proteins to the promoter site of *recA*, *lexA*, and the reporter gene are denoted by 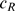, 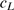, and 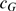, respectively, while their dissociation rate constants are denoted by 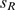, 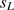, and 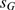, respectively. The rate constant for the binding of RecA and LexA proteins is denoted by 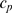. For simplicity, we assume that the concentration of each of the molecules is homogeneous throughout the cell. Also, we assume that transcriptional regulation is performed by a single regulator protein, namely there is no cooperative binding.

In Table 1 we present the rate constants of all the processes that take place in our model of the SOS system as well as in the reporter gene. Expressions for the actual rates of all processes, which depend on the copy numbers of the participating molecules, are also shown. In the rate equations, these copy numbers are given by real positive numbers. In the stochastic simulations these copy numbers are non-negative integers. For the sake of completeness, the detailed form of the rate equations is shown in the Supporting Information (Text S1).

The values of the rate constants that were used in Fig. 2 and Fig. 3 appear in Table 1. The number of plasmids in the simulation is 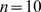, in accordance with the experimental procedure in Ref. [11]. The range of parameter that was considered was derived from the analysis of experimental results in *E. coli* [18], [28]–[32].

## Supporting Information

Text S1 The detailed rate equations for the model, describing the part of the SOS system that was used in the analysis.
(0.03 MB PDF)
