# Supplementary material for: Stochastic Analysis of the SOS Response in Escherichia coli
Source: PLoS One. 2009 May 8;4(5):e5363. doi: 10.1371/journal.pone.0005363 (PMC2675100; doi:10.1371/journal.pone.0005363)
Supplement: Text S1 — The detailed rate equations for the model, describing the part of the SOS system that was used in the analysis. (0.03 MB PDF) [file pone.0005363.s001.pdf]

Supporting information for the paper: Stochastic Analysis of the SOS Response in *Escherichia coli*

Yishai Shimoni[1,2], Shoshy Altuvia[1], Hanah Margalit[1], and Ofer Biham[2]

[1] Department of Molecular Genetics and Biotechnology, Faculty of Medicine, The Hebrew University, Jerusalem 91120, Israel

[2] Racah Institute of Physics, The Hebrew University, Jerusalem 91904, Israel

I. RATE EQUATIONS FOR THE SOS MODEL

Below we introduce the notation for the SOS model. The number of reporter genes is denoted by n. We denote the number of *recA* mRNAs, *lexA* mRNAs, and reporter gene mRNAs by m_R, m_L, and m_G, respectively. We denote the number of RecA, LexA, and GFP proteins by R, L, and G, respectively. The number of LexA proteins that are bound to the promoter site of *recA*, *lexA*, and the reporter gene are denoted by b_R, b_L, and b_G, respectively. For convenience, we also use the number of free promoter sites of each gene, denoting them by f_i, $i = L$,R or G. The rate constant for the transcription of *recA* mRNAs, *lexA* mRNAs, and the reporter gene mRNAs are denoted by g_{m_R}, g_{m_L}, and g_{m_G}, respectively. The translation rate constants of the RecA, LexA, and GFP proteins are denoted by g_R, g_L, and g_G, respectively. The degradation rate constants are denoted by d_j, where $j = m_R, m_L, m_G$ for mRNA molecules and $j = R, L$, and G for proteins. The rate constant for the binding of LexA proteins to the promoter site of *recA*, *lexA*, and the reporter gene are denoted by c_R, c_L, and c_G, respectively, while their dissociation rate constants are denoted by s_R, s_L, and s_G, respectively. The rate constant for the binding of RecA and LexA proteins is denoted by c_p. We note that when RecA proteins mark LexA for cleavage, RecA proteins remain unaffected. The rate equations for the SOS model take the form

$$\frac{dm_R}{dt} = g_{m_R}(1 - b_R) - d_{m_R} m_R, \quad (1)$$

$$\frac{dm_L}{dt} = g_{m_L}(1 - b_L) - d_{m_L} m_L, \quad (2)$$

$$\frac{dR}{dt} = g_R m_R - d_R R, \quad (3)$$

$$\begin{aligned}\frac{dL}{dt} &= g_L m_L - d_L L - c_L(1 - b_L)L + s_L b_L - c_R(1 - b_R)L + s_R b_R \\ &- c_G(n - b_G)L + s_G b_G - c_p R L,\end{aligned} \quad (4)$$

$$\frac{db_R}{dt} = c_R(1 - b_R)L - s_R b_R, \quad (5)$$

$$\frac{db_L}{dt} = c_L(1 - b_L)L - s_L b_L, \quad (6)$$

$$\frac{dm_G}{dt} = g_{m_G}(n - b_G) - d_{m_G} m_G, \quad (7)$$

$$\frac{dG}{dt} = g_G m_G, \quad (8)$$

$$\frac{db_G}{dt} = c_G(n - b_G)L - s_G b_G. \quad (9)$$
